# The association between childhood obesity and major adverse liver outcomes in adolescence and young adulthood

**DOI:** 10.1016/j.jhepr.2025.101425

**Published:** 2025-04-11

**Authors:** Resthie R. Putri, Thomas Casswall, Pernilla Danielsson, Claude Marcus, Emilia Hagman

**Affiliations:** 1 Department of Clinical Science, Intervention and Technology, Karolinska Institutet, Stockholm, Sweden; 2 Department of Medical Epidemiology and Biostatistics, Karolinska Institutet, Stockholm, Sweden

**Keywords:** obesity, paediatric obesity, liver failure, cirrhosis, alcohol use disorder, type 2 diabetes

## Abstract

**Background & Aims:**

Paediatric obesity is associated with liver steatosis and injury. We aimed to investigate the association between paediatric obesity and the risk of major adverse liver outcomes (MALOs) during adolescence and adulthood.

**Methods:**

A cohort study of children with overweight or obesity enrolled in the Swedish Childhood Obesity Treatment Register (1997–2020) was performed (n = 29,321). Controls from the general population matched by sex, birth year, and resident areas were obtained (n = 141,510). The individuals were followed from age 10 (or obesity treatment initiation) up to age 40. MALOs were defined as any occurrence of cirrhosis, hepatocellular carcinoma, oesophageal or gastric varices, portal hypertension, liver transplantation, ascites, liver failure, or liver-related mortality.

**Results:**

During a median follow-up of 8.3 [Q1–Q3: 5.5–11.8] years, MALOs were identified in 77 individuals. The cumulative incidence of MALOs by age 40 was 1.14% in the obesity cohort and 0.52% in the control group. Childhood adiposity was associated with the risk of MALOs (hazard ratio 2.15, 95% CI 1.33–3.48, *p =* 0.002). Individuals who had childhood obesity and developed alcohol use disorder during follow-up had an even higher risk of MALOs than controls without alcohol use disorder (hazard ratio 7.64, 95% CI 2.73–21.47, *p* <0.001). Type 2 diabetes did not mediate the association between childhood obesity and MALOs (*p =* 0.54).

**Conclusions:**

Paediatric obesity is associated with a two-fold increased risk of MALOs. However, the absolute risk of developing MALOs by age 40 remains low.

**Impact and implications:**

Firstly, healthcare providers should recognise that the consequences of paediatric obesity are not limited to immediate health concerns but rather present a sustained consequence on liver health into adulthood. Secondly, our findings revealed that a substantial proportion of individuals with alcohol use disorder experienced onset during adolescence, significantly amplifying the risk of major adverse liver outcomes. This underscores the importance of incorporating routine assessment for alcohol use disorder within paediatric care, particularly during adolescence, to identify and mitigate this increased risk. Thirdly, while the incidence of major adverse liver outcomes up to age 40 remains low, we identify a population at increased risk. This could help to refine risk stratification and target interventions more effectively.

## Highlights

• Paediatric obesity is associated with a twofold higher risk of major adverse liver outcomes.
• By age 40, 1.1% of the paediatric obesity cohort had developed major adverse liver outcomes.
• Paediatric obesity enhanced the risk in individuals who had alcohol use disorders during adolescence or young adulthood.

## Graphical abstract

**Figure:**
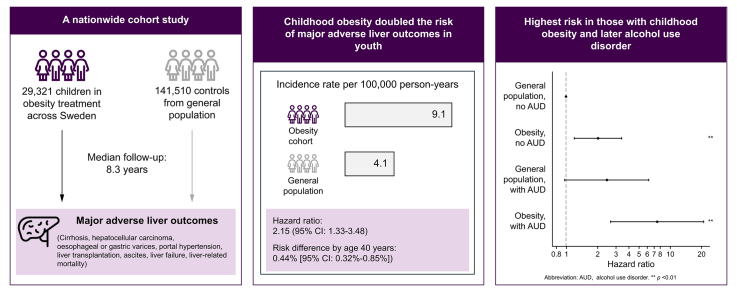
The figure illustrates the cumulative incidence of major adverse liver outcomes was higher in the obesity cohort than in the general population comparators, with a hazard ratio of 2.15 (95% CI: 1.33–3.48, *p* = 0.002).

## Introduction

Metabolic dysfunction associated steatotic liver disease (MASLD) affects an estimated half of children with obesity.^1^ Among those with MASLD, 10-20% exhibit advanced fibrosis. Given the high prevalence of MASLD in paediatric obesity, it is plausible that the risk of major adverse liver outcomes (MALOs) in early adulthood would be substantial. However, it is not yet established whether paediatric obesity contributes to increased future risk of MALOs. A recent review highlights the urgent need for longitudinal research to understand the risk of liver progression in the population with paediatric obesity.^2^ In adults, a large population-based cohort showed a positive association between high body mass index (BMI) at age 18-19 years and the risk of severe liver disease.^3^ Moreover, a multicentre trial found adult obesity as a risk factor for decompensated cirrhosis, independently of the cause of liver disease.^4^ This study aimed to investigate the association between paediatric obesity and the risk of MALOs during adolescence and adulthood.

## Patients and methods

### Study design, participants, and setting

This was a cohort study of children undergoing obesity treatment and enrolled in the Swedish Childhood Obesity Treatment Register (BORIS, e-boris.se/in-english/) (year 1997– 2020). During the study period, all patients received primarily lifestyle-based obesity treatment. The register covers paediatric obesity treatment at all healthcare levels across Sweden. Both patients with and without obesity-related morbidities are recorded in the register.

Included in this study were children with overweight or obesity according to the International Obesity Task Force^5^ at treatment initiation. Controls from the general population were paired (ratio 1:5, without replacement) based on sex, birth year, and residential area. Exclusion criteria were individuals who had MALOs before the age of 10 years, and individuals with genetic syndromes associated with obesity or MALOs (*i.e*., Alagille, Down, Fragile X, Klinefelter, Laurence-Moon-Biedl, Noonan, Prader–Willi, Silver-Russel, Turner syndrome; see Table S1).

The study population was followed from the index date until the outcome of MALOs, death, emigration, age of 40 years, or the end of follow-up (July 2023), whichever came first (diagnostic codes for MALOs in Table S1). The index date for the obesity cohort was the date when the individuals turned 10 years of age, or the date of entering paediatric obesity treatment if the individuals were older than 10 when entering obesity treatment. The controls from the general population inherited the index date from their matched case in the obesity cohort.

### Variables

• The main exposure was excess adiposity based on BMI threshold according to the International Obesity Task Force.^5^ Within the obesity cohort, the degree of adiposity was defined based on their initial BMI standard deviation score (SDS) for sex and age, and categorised as overweight, obesity class I, class II, and class III.^5^^,^^6^
• The outcome was MALOs. This was a composite variable, defined as any diagnosis of cirrhosis, oesophageal varices, gastric varices, portal hypertension, ascites, liver failure, hepatocellular carcinoma (HCC), liver transplantation, or liver-related mortality according to the International Classification of Diseases 10^th^ Revision (ICD10) (ICD-10 codes for the outcome in Table S1). An individual could have more than one of the diagnoses.
• Alcohol use disorder during follow-up was assessed for its potential synergistic effect with obesity on increasing the risk of MALOs. Alcohol use disorder was defined as any diagnosis related to alcohol use (*e.g.*, mental disorder due to the use of alcohol, alcohol-induced gastritis, alcohol-induced pancreatitis, alcohol-related liver disease, alcohol-related polyneuropathy) (ICD-10 codes for alcohol use disorder in Table S2).
• Type 2 diabetes which occurred during follow-up was assessed as a potential mediator given that type 2 diabetes has been indicated to lie in the causal pathway between obesity and MALOs in adult studies.^3^^,^^7^ Type 2 diabetes was defined as the presence of diagnosis and/or medications for type 2 diabetes (ICD-10 codes in Table S3) based on an algorithm (Fig. S1).^8^
• Metabolic bariatric surgery during follow-up (codes in Table S3) was considered as a competing risk in a sensitivity analysis, given that weight reduction after the surgery may improve hepatic steatosis and thus alter the risk of developing MALOs.^9^

### Data source

The main data source was BORIS, which records clinical and laboratory visits in paediatric obesity centres across Sweden.^10^ To date, more than 120 paediatric centres, ranging from primary care to university hospitals, providing obesity care across the country have registered their patients in BORIS.

Everyone residing in Sweden has a unique personal identity number. The personal identity number was used to link data from various national registers. The Total Population Register (1997–2020) was utilised to obtain general population comparators for the obesity cohort and to obtain data on emigration. Additionally, Swedish national registers containing medical data were utilised. Firstly, the National Patient Register (1997–2023 for inpatient register and 2001–2023 for outpatient register) was used to obtain diagnoses of MALOs, alcohol use disorder, type 2 diabetes, diseases included as exclusion criteria, and metabolic bariatric surgery. The National Patient Register records all medical diagnoses in inpatient and specialised outpatient care in the country. In general, the positive predictive value of most diagnoses in this register is >85%.^11^ For liver-related diagnoses, the positive predictive value for cirrhosis is 91%, for ascites in combination with a code for chronic liver disease is 93%, yet for ascites only is 43%.^12^ Secondly, the Cause of Death Register (1997–2023) was utilised to identify the occurrence and date for liver-related mortality and mortality from other causes. Thirdly, the Prescribed Drug Register (2005–2023) was used to identify the prescription of antidiabetic medications.

### Statistical analysis

Descriptive statistics were reported as proportions for categorical variables and median [Q1–Q3] for continuous variables. The incidence rate (IR) for MALOs per 100,000 person-years (P–Y) was calculated.

To assess the effect of paediatric obesity on the risk of developing MALOs, a flexible parametric survival model was performed. The event of interest was MALOs. Non-liver-related mortality was considered a competing risk. Age in years was used as a timescale. The cumulative incidence of MALOs between age 10 and 40 years in the obesity cohort and the general population comparators was estimated. An unadjusted model and a model adjusted for sex and alcohol use disorder were performed. The proportional hazard assumption was met according to Schoenfeld residuals. The longitudinal change of BMI SDS over time within the obesity cohort was estimated using a linear mixed model incorporating age at the measurement of BMI SDS in the model.

The joint effect between paediatric obesity and alcohol use disorder during follow-up on the risk for MALOs was quantified in additive scale.^13^ Hazard ratios (HRs) were estimated using Cox regression. The presence of a synergistic effect was measured using relative excess risk due to interaction, attributable proportion, and synergy index.

Mediation analysis based on a counterfactual framework^14^ was performed to decompose the association between paediatric obesity (exposure) and MALOs (outcome) into an association mediated by type 2 diabetes (indirect effect) and an association not mediated by the mediator (direct effect). The proportion of the association mediated by type 2 diabetes was also estimated.

Two different sensitivity analyses were performed. Firstly, patients with ascites but no codes for any chronic liver disease were excluded from the analysis. Secondly, an analysis by adding metabolic bariatric surgery as a competing risk was performed.

## Results

A total of 29,321 individuals from the obesity cohort and 141,510 matched controls from the general population were included in the analysis. The baseline characteristics of the population are described in Table 1.

**Table 1:** Baseline characteristics.

	Childhood obesity cohort	Matched general population comparators^1^
	(n = 29,321)	(n = 141,510)
Sex		
Boys, n (%)	15,548 (53.0)	75,207 (53.1)
Girls, n (%)	13,773 (47.0)	66,303 (44.9)
Age at baseline, median (Q1–Q3)	10.3 (10.0–13.0)	10.4 (10.0–13.0)
BMI SDS at baseline, median (Q1–Q3)	2.75 (2.47–3.10)	
Overweight, n (%)	2,744 (9.4)	
Obesity class I, n (%)	14,873 (50.7)	
Obesity class II, n (%)	7,407 (25.3)	
Obesity class III, n (%)	4,297 (14.6)	
End of follow-up		
MALO^2^, n (%)	24 (0.08)	53 (0.04)
Mortality from non-liver causes, n (%)	70 (0.24)	198 (0.14)
Emigration, n (%)	233 (0.79)	2,204 (1.56)
Age at MALO diagnosis, median (Q1–Q3)	21.6 (17.3–26.5)	20.5 (17.0–25.0)
Type 2 diabetes, n (%)	1,311 (4.47)	339 (0.24)
Alcohol use disorder during follow-up, n (%)	631 (2.15)	2,735 (1.93)

BMI, body mass index; MALO, major adverse liver outcome; SDS, standard deviation score.

Continuous variables are presented as median (Q1–Q3), whereas categorical variables are presented as number and proportion (%).

1 Controls from the general population were matched by sex, birth year, and resident areas.

2 No liver-related mortality was observed during the follow-up period.

### Paediatric obesity was associated with an increased risk of MALOs

**Fig. 1:**
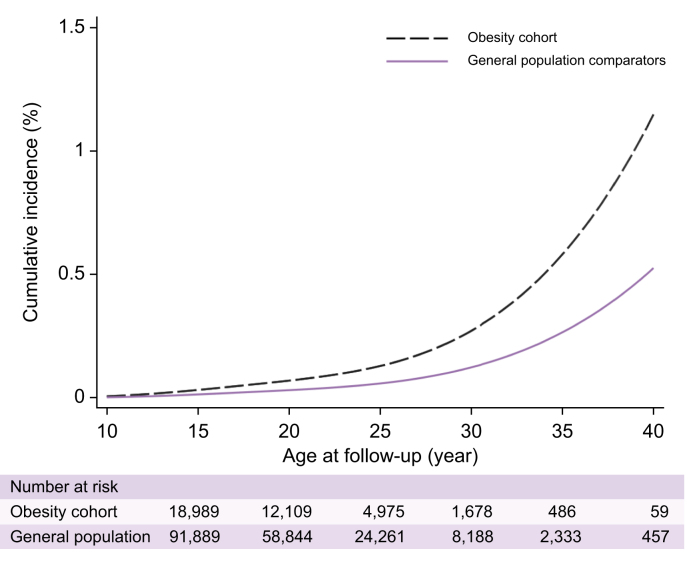
Cumulative incidence of MALOs between age 10 and 40 years in the obesity cohort and the general population comparators. Cumulative incidence in each group was estimated using a flexible parametric survival model. MALOs, major adverse liver outcomes.

During 1,551,994 P–Y of follow-up (median length of follow-up: 8.3 [Q1–Q3 5.5–11.8] years; median age at the end of follow-up: 18.8 [Q1–Q3 15.2–23.0] years), MALOs were observed in 24 individuals in the obesity cohort (IR: 9.0 per 100,000 P–Y) and 53 individuals (IR: 4.1 per 100,000 P–Y) in the general population comparators. The obesity cohort had a higher incidence of MALOs over time (Fig. 1), with an estimated cumulative incidence of 1.14% in the obesity cohort and 0.52% in the general population comparators by age 40 (estimated risk difference: 0.44%, 95% CI 0.32–0.85%). The incidence of liver failure and other cirrhosis-related outcomes, separately, is presented in Fig. S2. Paediatric obesity was associated with an increased risk of MALOs (HR 2.18, 95% CI 1.35–3.53, *p =* 0.001). The risk was similar after adjustment for sex and alcohol use disorder (HR 2.15, 95% CI 1.33–3.48, *p =* 0.002).

Of all individuals developing MALOs in this study (n = 77), the most common diagnosis was ascites (40.3%, n = 31), followed by acute or subacute liver failure (23.4%, n = 18), chronic liver failure (18.2%, n = 14), cirrhosis (10.4%, n = 8), oesophageal or gastric varices (10.4%, n = 8), post-liver transplantation (9.1%, n = 7), and portal hypertension (7.8%, n = 6). No occurrence of hepatocellular carcinoma or liver-related mortality was observed.

Within the obesity cohort, compared to those who did not develop the outcome, the group with MALOs had a higher proportion of class II or III obesity at baseline (66.7% among individuals with MALOs *vs.* 38.9% among those without MALOs) and a lower proportion of obesity remission (8.3% *vs.* 16.6%) (Table S4). Similarly, BMI SDS over time during paediatric years was higher in individuals with MALOs than those without MALOs (in the non-MALO group: average BMI SDS at age 10 years = 2.63 and at age 17 years = 2.64; in MALO group: average BMI SDS at age 10 years = 2.80 and at age 17 years = 3.27) (Fig. S3). Moreover, individuals who had a MASLD diagnosis had a higher incidence of MALOs over time compared to those without a MASLD diagnosis (*p <*0.001). Likewise, alcohol use disorder in the obesity cohort was also associated with a higher incidence of MALOs (*p =* 0.019). The incidence of MALOs did not differ by the initial obesity class (*p =* 0.404). The incidence of MALOs by MASLD, alcohol use disorder, and initial obesity class is shown in Fig. S4.

### The joint effect of paediatric obesity and alcohol use disorders during follow-up

**Fig. 2:**
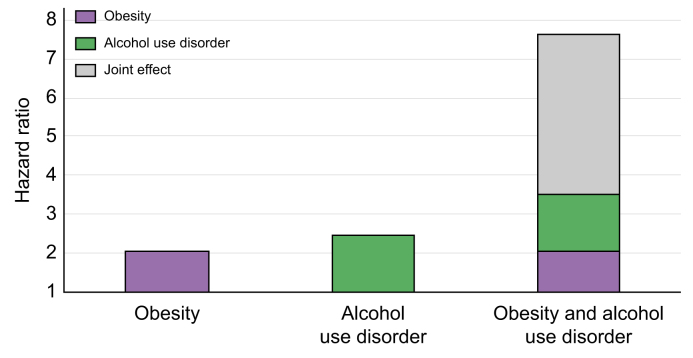
Joint effect between obesity and alcohol use disorder on the risk of MALOs. Hazard ratios were estimated using Cox regression. The reference group (hazard ratio = 1) was general population comparators without any diagnosis of alcohol use disorders. MALOs, major adverse liver outcomes.

Fig. 2 shows HRs of developing MALOs, with the general population without a diagnosis of alcohol use disorder as the reference. The highest HR was observed in the group with obesity and alcohol use disorder (HR 7.64, 95% CI 2.73–21.47, *p* <0.001). Measures of additive interaction showed a relative excess risk due to interaction of 4.11 (95% CI -3.79 to 12.01, *p =* 0.31), attributable proportion of 0.54 (95% CI -0.008 to 1.09, *p =* 0.053), and synergy index of 2.64 (95% CI 0.60–11.59, *p =* 0.20).

### The effect of paediatric obesity on MALOs was not mediated by type 2 diabetes

In the mediation analysis, a direct effect of paediatric obesity on MALOs was observed (HR 2.16, 95% CI 1.09–3.24, *p =* 0.034), whereas no significant mediating effect of type 2 diabetes was found (indirect effect; HR 1.07, 95% CI -1.15 to 1.30, *p =* 0.528). The proportion of the association mediated by type 2 diabetes was 6.3% (95% CI -13.8% to 26.3%, *p =* 0.539).

### Sensitivity analysis

The first sensitivity analysis, excluding ascites without any diagnosis of chronic liver disease from the MALO outcome, showed a persistent association between paediatric obesity and MALOs (HR 2.65, 95% CI 1.47–4.79, *p =* 0.001). In the second sensitivity analysis, adding metabolic bariatric surgery as one of the competing risks, the association between paediatric obesity and MALOs also remained (HR 2.20, 95% CI 1.34–3.59, *p =* 0.002).

## Discussion

This nationwide cohort study demonstrates an association between paediatric obesity and increased risk for MALOs during adolescence and young adulthood. The association was not mediated by type 2 diabetes. Furthermore, a synergistic effect between childhood obesity and alcohol use disorder on MALOs was observed during follow-up.

In line with findings in adult studies,^3^^,^^15^ we showed that obesity in children and adolescents was associated with double the relative risk for MALOs. While the previous studies^3^^,^^15^ showed that adult obesity was associated with increased risk in middle and late adulthood, the current study suggests that excess adiposity starting in childhood may contribute to the progression of MALOs and earlier onset of MALO occurrence. However, the double relative risk found in this study should be interpreted carefully given that the absolute risk difference was relatively small.

Despite the relatively low cumulative incidence of MALOs in the obesity cohort during paediatric years, a substantial increasing trend of MALO incidence after age 20 years was observed in the current study. A longer follow-up time would raise the cumulative incidence, given the long-term nature of liver disease progression.^16^ Most cases of MALOs during adulthood today occur between age 50 and 80 years.^17^ Although less frequent in adolescence and young adulthood, MALOs (for instance, cirrhosis) are generally irreversible, often lead to premature mortality or liver transplantation,^18^ and are associated with a high healthcare burden.^19^ Moreover, if the prevalence of childhood obesity continues to rise, it is likely that the total number of patients with MALOs will increase and affect healthcare decision-making. Targeted interventions are warranted in patients at a high risk of MALOs. Further studies are crucial to identify subgroups in the paediatric obesity population at the greatest risk of developing MALOs.

The reasons for the positive association between paediatric obesity and the risk of MALOs remain uncertain. A possible explanation is that paediatric obesity is strongly associated with MASLD, which can progress to cirrhosis.^20^ Nevertheless, among patients with paediatric obesity who developed MALOs in the present study, about 15% of them had chronic liver diseases other than MASLD, including chronic viral hepatitis, autoimmune hepatitis, primary biliary cholangitis, and alcohol-related liver disease. Hence, it is also possible that low-grade inflammation induced by paediatric obesity^21^ may contribute to the progression of chronic liver disease, regardless of the cause. In addition, insulin resistance, linked with paediatric obesity, may promote inflammatory pathways in the liver and lead to hepatic inflammation and apoptosis.^22^ Proinflammatory cytokine levels may be elevated further by the crosstalk between the liver and other organs in an environment of excess adiposity.^22^ Another explanation is that adult obesity might be a mediator of the association given that paediatric obesity is likely to persist to adulthood.^23^ In addition, the current study also showed that alcohol use disorder during follow-up is more common in the obesity cohort than the general population comparators. Although adjustment for alcohol use disorder was performed in analysing the association between paediatric obesity and MALOs, the actual proportion of individuals with excessive alcohol consumption was unknown and could potentially contribute to the association.

The current study suggests a potential synergistic effect of childhood obesity and alcohol use disorders during follow-up on the increased risk of MALOs. Likewise, a Swedish register-based study in adults also found that co-occurrence of MASLD and alcohol use disorder is associated with a 5-fold greater risk of MALOs than MASLD only.^24^ In the present study, about 25% of the individuals with alcohol use disorders had already had the diagnosis before 18 years of age. While the traditional approach usually assumes a single cause of liver disease (*e.g*. non-alcohol- *vs.* alcohol-related liver disease), it is recommended to consider mixed causes, especially between metabolic dysfunction and alcohol-related liver disease, even in the paediatric population. The recent international consensus statement defined a new category named MetALD^25^ to describe an overlap condition between MASLD and alcohol-related liver disease. The possibility of such an overlapping condition is also emphasized by paediatric hepatology societies.^26^ Differentiating solely MASLD from MASLD combined with alcohol use disorder may benefit clinical practice because the latter seems to be associated with a higher risk of developing MALOs. Thus, it is important for paediatricians to assess alcohol use disorder, especially in caring for adolescents with obesity.

While adult studies have observed type 2 diabetes to be a major contributor, rather than solely obesity, to liver disease progression,^27^^,^^28^ such a finding was not seen in the current study. The mediation analysis found that the excess risk of MALOs attributable to paediatric obesity was greater than that of type 2 diabetes, indicating that paediatric obesity plays a more important role in the risk of MALOs during adolescence and young adulthood than type 2 diabetes. In addition, we did not observe a significant mediating effect of type 2 diabetes in the association between paediatric obesity and MALOs. A possible explanation is that paediatric obesity generally precedes type 2 diabetes, leading to a longer duration of obesity exposure in the liver. Further, a prolonged duration of type 2 diabetes may also be necessary to affect MALO development.

To our knowledge, this is the first nationwide study with a long follow-up attempting to investigate the effect of childhood obesity on the development of MALOs. The registers used in this study cover the whole nation. MALOs typically require specialised care, and all diagnoses in inpatient and specialised outpatient care in Sweden are recorded in the patient register. Thus, the internal validity of the study is strengthened. However, this study has some limitations. Firstly, data on the underlying cause of MALOs are lacking. Secondly, weight status in the general population comparators and weight trajectories of the obesity cohort after paediatric years were unknown. However, most children in BORIS still had obesity at their last childhood obesity treatment.^29^ Future studies incorporating adult weight trajectories are warranted to unravel the mechanism linking paediatric obesity to MALOs. Thirdly, the characteristics of MALOs in this study may not be typical as MALOs usually occur in older age groups. Fourthly, the result of joint effect and mediation analyses might be susceptible to chance findings given the very limited number of individuals with the outcome. Furthermore, the precision and statistical power of the mediation analysis and stratified analyses might be limited due to the small sample size of individuals experiencing the outcome. In addition, the external validity of the findings may be limited to populations with similar demography as Sweden. Different genetic liability, patterns of alcohol consumption and sugar intake between countries may affect the generalisability of the findings. For example, Asian countries, characterised generally by lower alcohol consumption but higher sugar-sweetened beverage intake compared to Western nations, illustrate how dietary factors can affect the risk of MALO.^30^^,^^31^ Lastly, some cases of liver disease with unspecific symptoms and alcohol use disorder might be underreported in the patient register.

Childhood obesity is associated with an increased risk of MALOs during adolescence and young adulthood. However, relatively few individuals with childhood obesity are affected by MALOs before 40 years of age.

## Abbreviations

BMI, body mass index; BORIS, the Swedish Childhood Obesity Treatment Register; HCC, hepatocellular carcinoma; HR, hazard ratio; ICD, the International Classification of Diseases; IR, incidence rate; MALOs, major adverse liver outcomes; MASLD, metabolic dysfunction associated steatotic liver disease; P–Y, person-years; SDS, standard deviation score.

## Financial support

This study was supported by the Freemason Foundation for Children’s Welfare inStockholm, the foundation of Sällskapet Barnavård, the HRH Crown Princess Lovisa Society for Child Care, Anna-Lisa & Arne Gustafsson’s foundation, The 10.13039/501100018713Center for Innovative Medicine (10.13039/501100018713CIMED). The funding sources had no involvement in study design, data analysis, data interpretation, manuscript writing, or the decision to submit the article.

## Authors’ contributions

Study conception and design: RRP, EH, and CM. Data curation: EH. Data analysis: RRP. Original draft preparation: RRP. TC, PDL, CM, EH, and RRP contributed to the interpretation of the results, provided critical feedback, and approved the final version of the manuscript.

## Data availability statement

Patient-level data cannot be shared publicly because of third-party data. Given that an ethical approval is obtained, any individual may apply for data from Statistics Sweden via information@scb.se, the Swedish National Board of Health and Welfare via registerservice@socialstyrelsen.se, and the Swedish Childhood Obesity Treatment Register via http://www.e-boris.se/in-english/.

## Conflict of interest

PD: Honoraria for lectures: Nestlé; Leadership or fiduciary role on scientific/medical committee: Member of the steering committee for the Swedish Childhood Obesity Treatment Register, Chairman of a working group developing the Swedish national guidelines for paediatric obesity treatment, secretary of the Swedish Childhood Obesity Association. CM: Consulting fees: Novo Nordisk, Rhythm, Oriflame Wellness, DeFaire Medical, Evira AB; Honoraria for lectures: Novo Nordisk, Nestlé, Oriflame Wellness, Astra Zeneca; Payment for expert testimony: Novo Nordic Foundation, Rhythm; Leadership or fiduciary role on scientific/medical committee: board member of ESPE Obesity working group, board member of the Swedish Pediatric Obesity Society, Register holder for the Swedish Childhood Obesity Treatment Register.

EH: Commissioned research for Novo Nordisk (2023), but not for the present study; Honoraria for lectures: Novo Nordisk and Nestlé; Leadership or fiduciary role on scientific/medical committee: Member of the steering committee for the Swedish Childhood Obesity Treatment Register. TC: Member of the working group within Swedish Society of Paediatric Gastroenterology, Hepatology, and Nutrition (SPGHAN) developing the Swedish national guidelines of paediatric MASLD.

RRP had no conflict of interest to disclose.

Please refer to the accompanying ICMJE disclosure forms for further details.
