# Supplementary material for: The association between childhood obesity and major adverse liver outcomes in adolescence and young adulthood
Source: JHEP Rep. 2025 Apr 11;7(7):101425. doi: 10.1016/j.jhepr.2025.101425 (PMC12205788; doi:10.1016/j.jhepr.2025.101425)
Supplement: Multimedia component 1 [file mmc1.pdf]

The association between childhood obesity and major adverse liver outcomes in adolescence and young adulthood

Resthie R Putri, Thomas Casswall, Pernilla Danielsson, Claude Marcus, Emilia Hagman

Table of contents

Table S1…………………………………………………………………………………..2

Table S2…………………………………………………………………………………..3

Table S3…………………………………………………………………………………..4

Table S4…………………………………………………………………………………..5

Fig. S1…………………………………………………………………………………….6

Fig. S2…………………………………………………………………………………….8

Fig. S3…………………………………………………………………………………….9

Fig. S4…………………………………………………………………………………...10

Table S1. ICD-10 codes for the outcome of major adverse liver outcomes (MALO) and the exclusion criteria of genetic syndromes

ICD-10 code	Diagnosis
MALO	
K74.6	Cirrhosis
K76.6	Portal hypertension
I86.4	Gastric varices
I85.0, I85.9, I98.2, I98.3	Oesophageal varices
R18	Ascites
K72.0, K72.1, K72.9	Liver failure
C22.0	Hepatocellular carcinoma
Z94.4	Liver transplantation
Genetic syndromes	
Q44.7	Alagille syndrome
Q90	Down syndrome
Q99.2	Fragile X syndrome
Q98	Klinefelter syndrome
Q87.8B	Laurence-Moon-Biedl syndrome
Q87.1E	Noonan syndrome
Q87.1F	Prader-Willi syndrome
Q87.1G	Silver-Russel syndrome
Q96	Turner syndrome

Table S2. ICD-10 codes for alcohol use disorder

ICD-10 code	Diagnosis
E24.4	Alcohol-induced pseudo-Cushing's syndrome
F10	Mental and behavioural disorders due to use of alcohol
G62.1	Alcoholic polyneuropathy
I42.6	Alcoholic cardiomyopathy
K29.2	Alcoholic gastritis
G31.2	Degeneration of nervous system due to alcohol
G72.1	Alcoholic myopathy
K70	Alcoholic liver disease
K85.2	Alcohol-induced acute pancreatitis
K86.0	Alcohol-induced chronic pancreatitis
T51	Toxic effect of alcohol

Table S3. Codes for diagnosis of type 2 diabetes, antidiabetic medications, and volume-restricting bariatric surgery

Data sources	Codes
Type 2 diabetes	
The Swedish National Patient Register (based on ICD-10 codes)	E11. Type 2 diabetes<br>O24.1. Pre-existing type 2 diabetes mellitus, in pregnancy, childbirth and the puerperium
The Prescribed Drug Register (based on Anatomical Therapeutic Chemical codes)	A10BA biguanides<br>A10BB sulfonylureas<br>A10BC sulfonamides<br>A10BD antidiabetic combinations<br>A10BF alpha glucosidase inhibitors<br>A10BG thiazolidinediones<br>A10BH DPP4 inhibitors<br>A10BJ GLP-1 analogues<br>A10BK SGLT2 inhibitors<br>Other antidiabetic A10BX<br>Insulin A10A
Metabolic bariatric surgery	
National Patient Register	Procedure code for volume-restricting bariatric surgery (JDF) in combination with ICD-10 code for obesity (E66).

Table S4. Characteristics of patients in the paediatric obesity cohort based on the event outcome occurrence of MALO

	No MALO (N = 28 287)	MALO (N = 24)
Age at treatment initiation, median (Q1, Q3)	10.5 (8.1, 13.2)	13.0 (9.2, 16.6)
Overweight or class I obesity, n (%)	17 273 (61.1)	8 (33.3)
Class II or III obesity, n (%)	11 014 (38.9)	16 (66.7)
Change in BMI SDS[1], median (Q1, Q3)	-0.06 (-0.31, 0)	-0.04 (-0.27, 0)
Stay in the same obesity class at the last visit of obesity treatment, n (%)	11 747 (68.4)	13 (68.4)
Obesity remission at the last visit of obesity treatment, n (%)	4692 (16.6)	2 (8.3)
Obesity treatment duration, median (Q1, Q3)	17.3 (3.3 – 41.0)	23.6 (8.4 – 54.9)
Any recorded ALT >35 U/L during obesity treatment[2], n (%)	4632 (25.2)	7 (36.9)
Any recorded fasting glucose ≥6.1 mmol/L during obesity treatment[3], n (%)	979 (5.5)	2 (10.5)

Abbreviations: ALT, alanine aminotransferases; BMI SDS, body mass index standard deviation score, Q1, quartile 1; Q3, quartile 3

[1]BMI SDS reduction was calculated among individuals who had more than 1 visit in obesity treatment as BMI SDS at the last visit – BMI SDS at the first visit.

[2]Among those who had at least one ALT data in the paediatric obesity register (n = 19 in the MALO group and n = 18395 in the non-MALO group)

[3]Among those who had at least one fasting glucose data in the paediatric obesity register (n = 19 in the MALO group and n = 17926 in the non-MALO group)

Fig. S1. Algorithm to ascertain type 2 diabetes

The algorithm is divided into three groups:

- Algorithm A: Individuals who had diagnosis of type 2 diabetes in specialized care (i.e., recorded in the Patient Register) but did not receive prescribed antidiabetic medications.
- Algorithm B: Individuals who received prescribed antidiabetic medications but did not have recorded diagnosis of type 2 diabetes in specialized care.
- Algorithm C: Individuals who both received antidiabetic medications and had recorded diagnosis of type 2 diabetes in specialized care.

Algorithm A: Diagnosis of type 2 diabetes in specialized care = yes & any antidiabetic medications = no

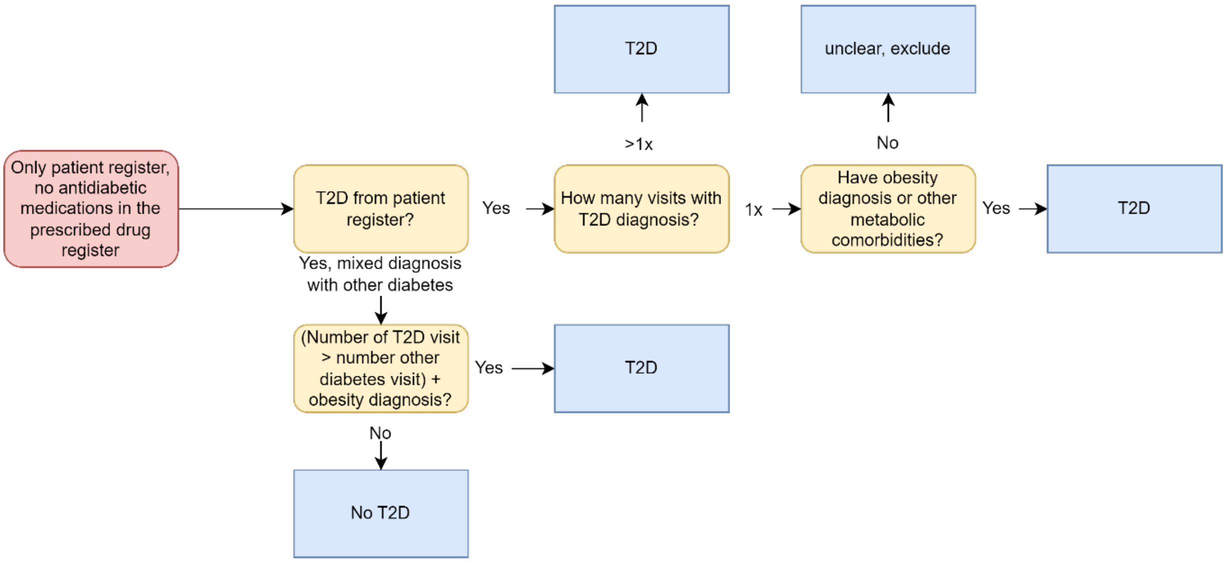

Algorithm B: Diagnosis of type 2 diabetes in specialized care = no & any antidiabetic medications = yes

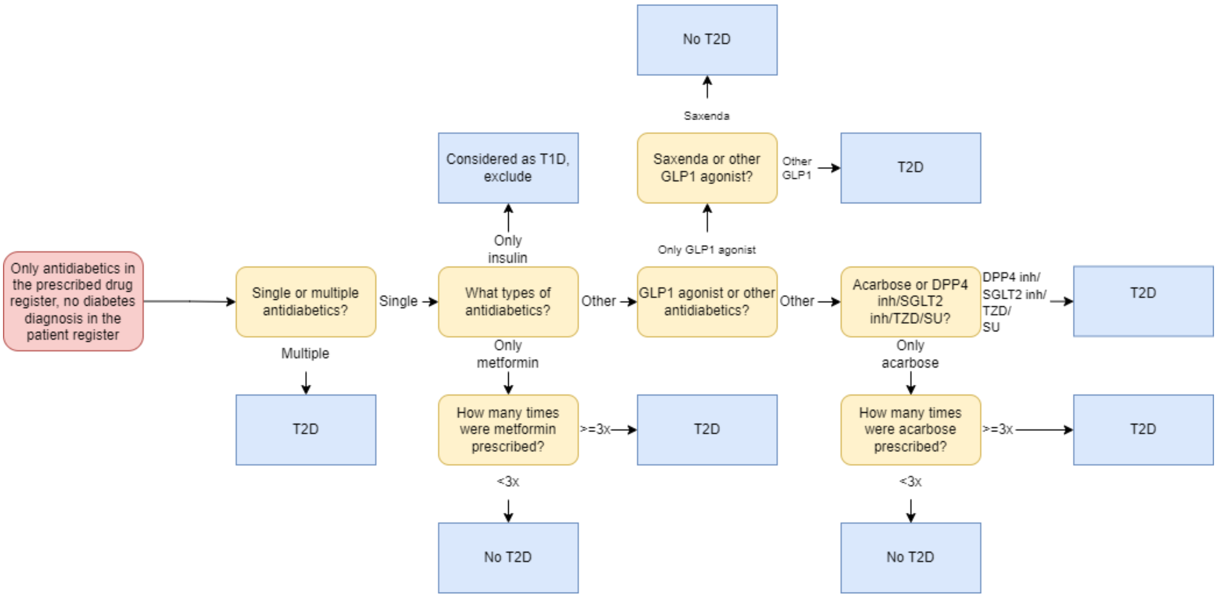

Algorithm C: Diagnosis of type 2 diabetes in specialized care = yes & any antidiabetic medications = yes

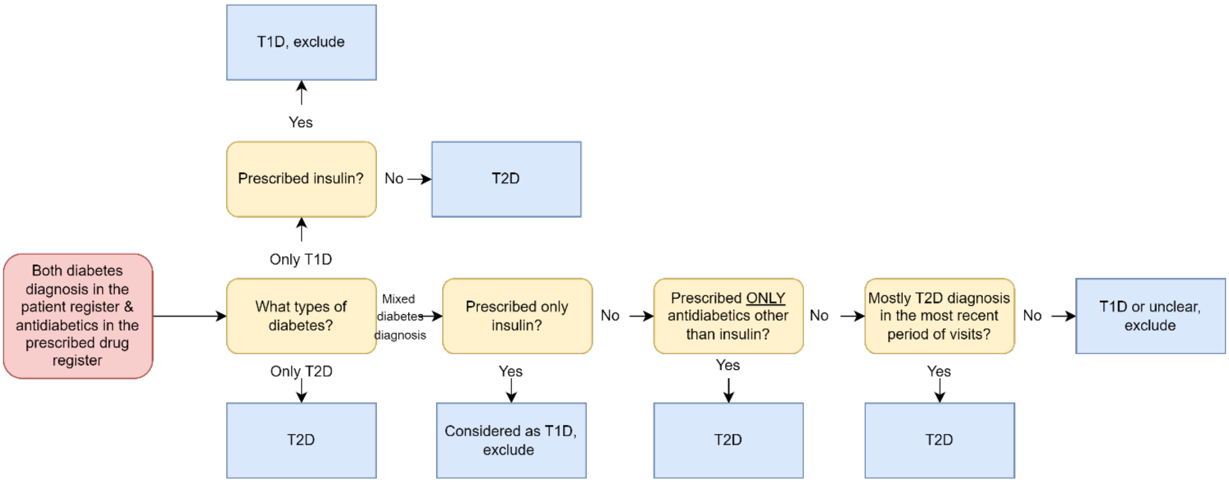

Fig. S2. Higher cumulative incidence of MALO (cirrhosis and liver failure, separately) in the paediatric obesity cohort and general population comparators

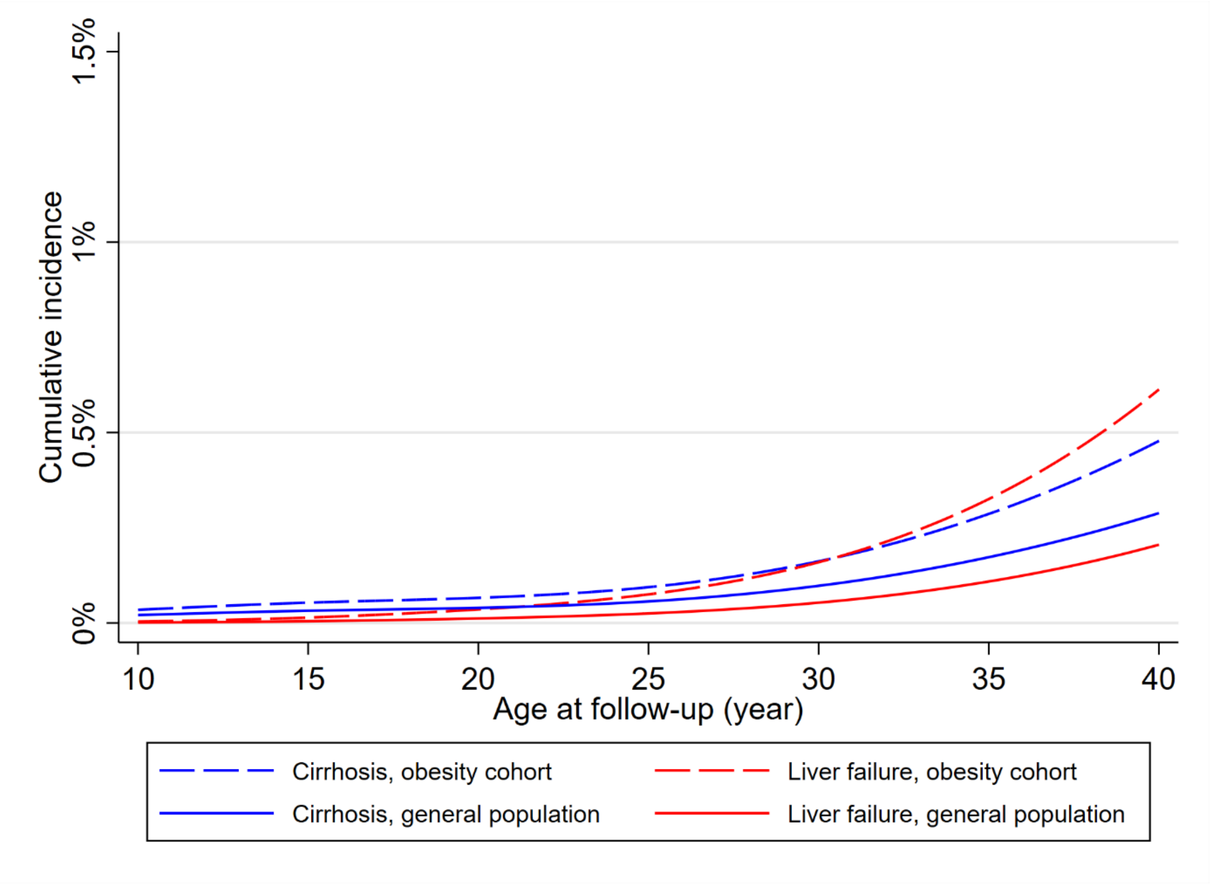

Estimated cumulative incidence of cirrhosis and liver failure was calculated based on flexible parametric models with competing risks with three degrees of freedom. Cirrhosis was defined based on all MALO related to decompensated cirrhosis (i.e., cirrhosis, oesophageal varices, gastric varices, portal hypertension, ascites). Liver failure was defined based on diagnosis of acute, sub-acute, or chronic liver failure.

Fig. S3. Longitudinal change of BMI SDS in the paediatric obesity cohort

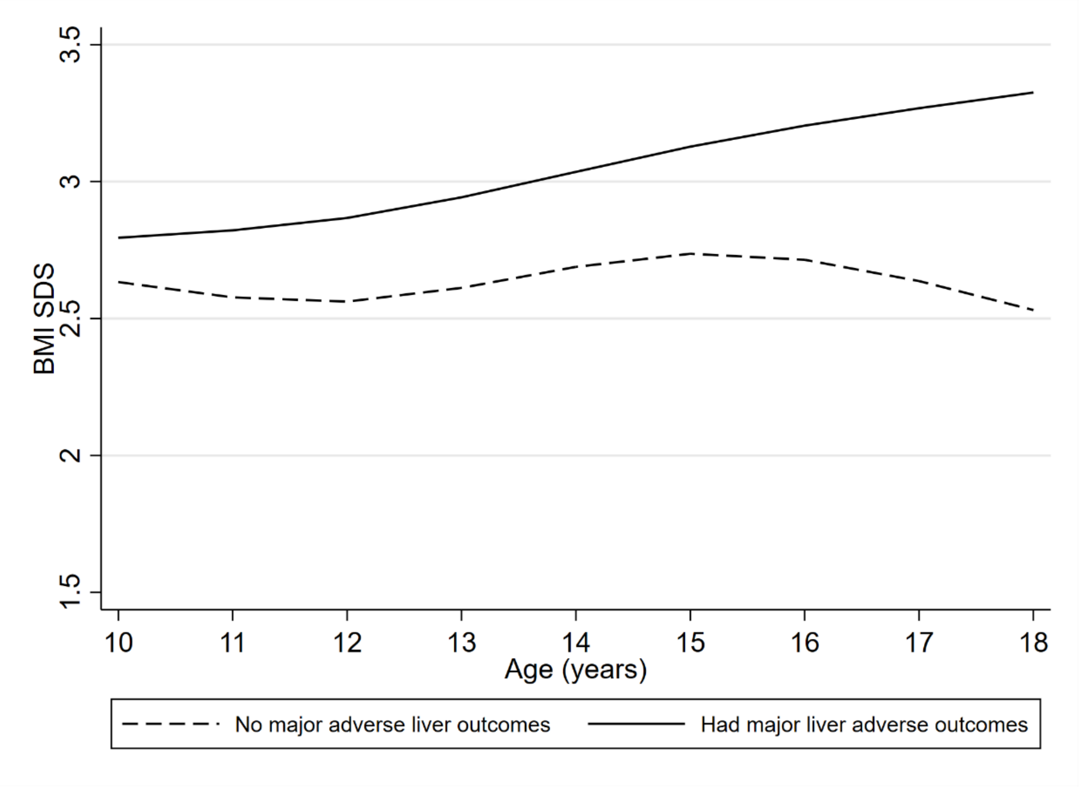

Y-axis represents estimated average BMI SDS. X-axis represents age (years). The longitudinal change of average BMI SDS over time was estimated using linear mixed model.

Fig. S4. Incidence of MALO within the obesity cohort, divided by diagnosis of MASLD, alcohol use disorder, and initial obesity class

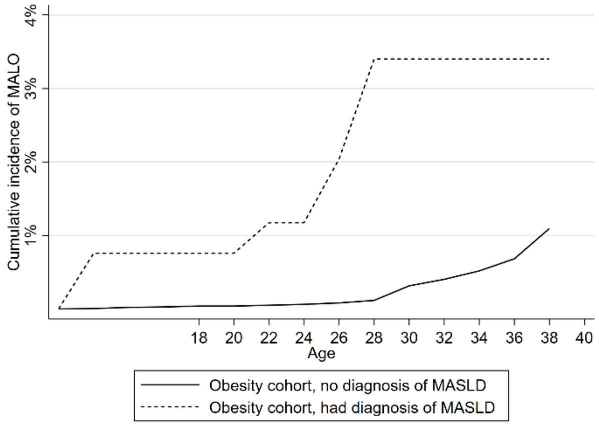

Figure S8 a) Incidence of MALO in the obesity cohort divided by diagnosis of MASLD, based on Kaplan Meier estimates (P log-rank <0.001).

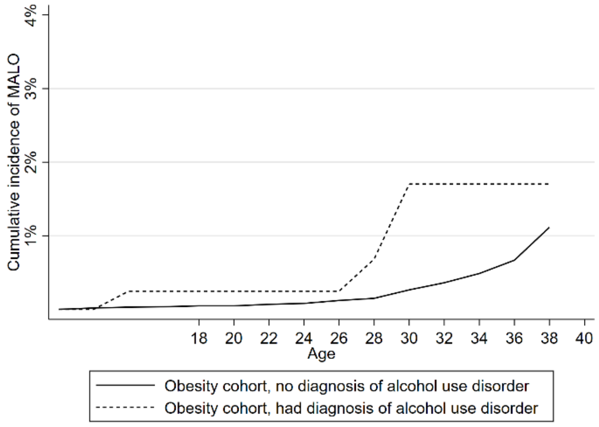

Figure S8 b) Incidence of MALO in the obesity cohort divided by diagnosis of alcohol use disorder, based on Kaplan Meier estimates (P log-rank = 0.019).

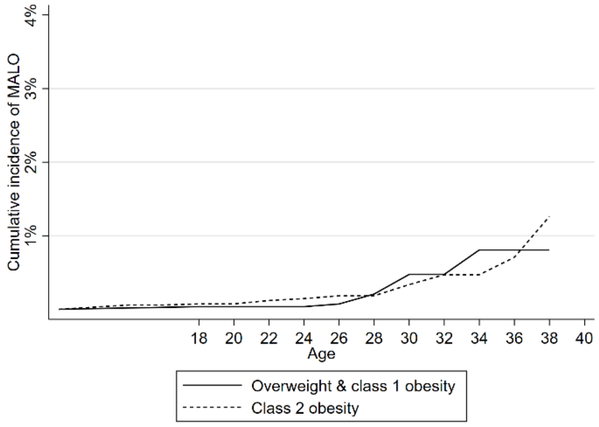

Figure S8 c) Incidence of MALO in the obesity cohort divided by initial degree of obesity, based on Kaplan Meier estimates (P log-rank = 0.404).
