# Supplementary material for: The association between childhood obesity and major adverse liver outcomes in adolescence and young adulthood
Source: JHEP Rep. 2025 Apr 11;7(7):101425. doi: 10.1016/j.jhepr.2025.101425 (PMC12205788; doi:10.1016/j.jhepr.2025.101425)
Supplement: Multimedia component 4 [file mmc4.pdf]

The association between childhood obesity and major adverse liver outcomes in adolescence and young adulthood

Resthie R. Putri[1,2,*], Thomas Casswall[1], Pernilla Danielsson[1], Claude Marcus[1], Emilia Hagman[1]

JHEP Reports **2025**. vol. 7 | 1–6

Check for updates

Background & Aims: Paediatric obesity is associated with liver steatosis and injury. We aimed to investigate the association between paediatric obesity and the risk of major adverse liver outcomes (MALOs) during adolescence and adulthood.

Methods: A cohort study of children with overweight or obesity enrolled in the Swedish Childhood Obesity Treatment Register (1997–2020) was performed (n = 29,321). Controls from the general population matched by sex, birth year, and resident areas were obtained (n = 141,510). The individuals were followed from age 10 (or obesity treatment initiation) up to age 40. MALOs were defined as any occurrence of cirrhosis, hepatocellular carcinoma, oesophageal or gastric varices, portal hypertension, liver transplantation, ascites, liver failure, or liver-related mortality.

Results: During a median follow-up of 8.3 [Q1–Q3: 5.5–11.8] years, MALOs were identified in 77 individuals. The cumulative incidence of MALOs by age 40 was 1.14% in the obesity cohort and 0.52% in the control group. Childhood adiposity was associated with the risk of MALOs (hazard ratio 2.15, 95% CI 1.33–3.48, p = 0.002). Individuals who had childhood obesity and developed alcohol use disorder during follow-up had an even higher risk of MALOs than controls without alcohol use disorder (hazard ratio 7.64, 95% CI 2.73–21.47, p <0.001). Type 2 diabetes did not mediate the association between childhood obesity and MALOs (p = 0.54).

Conclusions: Paediatric obesity is associated with a two-fold increased risk of MALOs. However, the absolute risk of developing MALOs by age 40 remains low.

© 2025 The Author(s). Published by Elsevier B.V. on behalf of European Association for the Study of the Liver (EASL). This is an open access article under the CC BY license (http://creativecommons.org/licenses/by/4.0/).

Introduction

Metabolic dysfunction associated steatotic liver disease (MASLD) affects an estimated half of children with obesity.[1] Among those with MASLD, 10-20% exhibit advanced fibrosis. Given the high prevalence of MASLD in paediatric obesity, it is plausible that the risk of major adverse liver outcomes (MALOs) in early adulthood would be substantial. However, it is not yet established whether paediatric obesity contributes to increased future risk of MALOs. A recent review highlights the urgent need for longitudinal research to understand the risk of liver progression in the population with paediatric obesity.[2] In adults, a large population-based cohort showed a positive association between high body mass index (BMI) at age 18-19 years and the risk of severe liver disease.[3] Moreover, a multicentre trial found adult obesity as a risk factor for decompensated cirrhosis, independently of the cause of liver disease.[4] This study aimed to investigate the association between paediatric obesity and the risk of MALOs during adolescence and adulthood.

Patients and methods

Study design, participants, and setting

This was a cohort study of children undergoing obesity treatment and enrolled in the Swedish Childhood Obesity Treatment Register (BORIS, e-boris.se/in-english/) (year 1997– 2020). During the study period, all patients received primarily lifestyle-based obesity treatment. The register covers paediatric obesity treatment at all healthcare levels across Sweden. Both patients with and without obesity-related morbidities are recorded in the register.

Included in this study were children with overweight or obesity according to the International Obesity Task Force[5] at treatment initiation. Controls from the general population were paired (ratio 1:5, without replacement) based on sex, birth year, and residential area. Exclusion criteria were individuals who had MALOs before the age of 10 years, and individuals with genetic syndromes associated with obesity or MALOs (*i.e.*, Alagille, Down, Fragile X, Klinefelter, Laurence-Moon-Biedl, Noonan, Prader–Willi, Silver-Russel, Turner syndrome; see Table S1).

* Corresponding author. Address: National Childhood Obesity Centre, Karolinska University Hospital, S-117 63 Stockholm, Sweden; +46760721447.
E-mail address: resthie.putri@ki.se (R.R. Putri).
https://doi.org/10.1016/j.jhepr.2025.101425

The study population was followed from the index date until the outcome of MALOs, death, emigration, age of 40 years, or the end of follow-up (July 2023), whichever came first (diagnostic codes for MALOs in Table S1). The index date for the obesity cohort was the date when the individuals turned 10 years of age, or the date of entering paediatric obesity treatment if the individuals were older than 10 when entering obesity treatment. The controls from the general population inherited the index date from their matched case in the obesity cohort.

Variables

- The main exposure was excess adiposity based on BMI threshold according to the International Obesity Task Force.[5] Within the obesity cohort, the degree of adiposity was defined based on their initial BMI standard deviation score (SDS) for sex and age, and categorised as overweight, obesity class I, class II, and class III.[5,6]
- The outcome was MALOs. This was a composite variable, defined as any diagnosis of cirrhosis, oesophageal varices, gastric varices, portal hypertension, ascites, liver failure, hepatocellular carcinoma (HCC), liver transplantation, or liver-related mortality according to the International Classification of Diseases 10th Revision (ICD10) (ICD-10 codes for the outcome in Table S1). An individual could have more than one of the diagnoses.
- Alcohol use disorder during follow-up was assessed for its potential synergistic effect with obesity on increasing the risk of MALOs. Alcohol use disorder was defined as any diagnosis related to alcohol use (*e.g.*, mental disorder due to the use of alcohol, alcohol-induced gastritis, alcohol-induced pancreatitis, alcohol-related liver disease, alcohol-related polyneuropathy) (ICD-10 codes for alcohol use disorder in Table S2).
- Type 2 diabetes which occurred during follow-up was assessed as a potential mediator given that type 2 diabetes has been indicated to lie in the causal pathway between obesity and MALOs in adult studies.[3,7] Type 2 diabetes was defined as the presence of diagnosis and/or medications for type 2 diabetes (ICD-10 codes in Table S3) based on an algorithm (Fig. S1).[8]
- Metabolic bariatric surgery during follow-up (codes in Table S3) was considered as a competing risk in a sensitivity analysis, given that weight reduction after the surgery may improve hepatic steatosis and thus alter the risk of developing MALOs.[9]

Data source

The main data source was BORIS, which records clinical and laboratory visits in paediatric obesity centres across Sweden.[10] To date, more than 120 paediatric centres, ranging from primary care to university hospitals, providing obesity care across the country have registered their patients in BORIS.

Everyone residing in Sweden has a unique personal identity number. The personal identity number was used to link data from various national registers. The Total Population Register (1997–2020) was utilised to obtain general population comparators for the obesity cohort and to obtain data on emigration. Additionally, Swedish national registers containing medical data were utilised. Firstly, the National Patient Register (1997–2023 for inpatient register and 2001–2023 for outpatient register) was used to obtain diagnoses of MALOs, alcohol use disorder, type 2 diabetes, diseases included as exclusion criteria, and metabolic bariatric surgery. The National Patient Register records all medical diagnoses in inpatient and specialised outpatient care in the country. In general, the positive predictive value of most diagnoses in this register is >85%.[11] For liver-related diagnoses, the positive predictive value for cirrhosis is 91%, for ascites in combination with a code for chronic liver disease is 93%, yet for ascites only is 43%.[12] Secondly, the Cause of Death Register (1997–2023) was utilised to identify the occurrence and date for liver-related mortality and mortality from other causes. Thirdly, the Prescribed Drug Register (2005–2023) was used to identify the prescription of antidiabetic medications.

Statistical analysis

Descriptive statistics were reported as proportions for categorical variables and median [Q1–Q3] for continuous variables. The incidence rate (IR) for MALOs per 100,000 person-years (P–Y) was calculated.

To assess the effect of paediatric obesity on the risk of developing MALOs, a flexible parametric survival model was performed. The event of interest was MALOs. Non-liver-related mortality was considered a competing risk. Age in years was used as a timescale. The cumulative incidence of MALOs between age 10 and 40 years in the obesity cohort and the general population comparators was estimated. An unadjusted model and a model adjusted for sex and alcohol use disorder were performed. The proportional hazard assumption was met according to Schoenfeld residuals. The longitudinal change of BMI SDS over time within the obesity cohort was estimated using a linear mixed model incorporating age at the measurement of BMI SDS in the model.

The joint effect between paediatric obesity and alcohol use disorder during follow-up on the risk for MALOs was quantified in additive scale.[13] Hazard ratios (HRs) were estimated using Cox regression. The presence of a synergistic effect was measured using relative excess risk due to interaction, attributable proportion, and synergy index.

Mediation analysis based on a counterfactual framework[14] was performed to decompose the association between paediatric obesity (exposure) and MALOs (outcome) into an association mediated by type 2 diabetes (indirect effect) and an association not mediated by the mediator (direct effect). The proportion of the association mediated by type 2 diabetes was also estimated.

Two different sensitivity analyses were performed. Firstly, patients with ascites but no codes for any chronic liver disease were excluded from the analysis. Secondly, an analysis by adding metabolic bariatric surgery as a competing risk was performed.

Results

A total of 29,321 individuals from the obesity cohort and 141,510 matched controls from the general population were included in the analysis. The baseline characteristics of the population are described in Table 1.

Table 1. Baseline characteristics.

	Childhood obesity cohort	Matched general population comparators[1]
	(n = 29,321)	(n = 141,510)
Sex		
Boys, n (%)	15,548 (53.0)	75,207 (53.1)
Girls, n (%)	13,773 (47.0)	66,303 (44.9)
Age at baseline, median (Q1–Q3)	10.3 (10.0–13.0)	10.4 (10.0–13.0)
BMI SDS at baseline, median (Q1–Q3)	2.75 (2.47–3.10)	
Overweight, n (%)	2,744 (9.4)	
Obesity class I, n (%)	14,873 (50.7)	
Obesity class II, n (%)	7,407 (25.3)	
Obesity class III, n (%)	4,297 (14.6)	
End of follow-up		
MALO[2], n (%)	24 (0.08)	53 (0.04)
Mortality from non-liver causes, n (%)	70 (0.24)	198 (0.14)
Emigration, n (%)	233 (0.79)	2,204 (1.56)
Age at MALO diagnosis, median (Q1–Q3)	21.6 (17.3–26.5)	20.5 (17.0–25.0)
Type 2 diabetes, n (%)	1,311 (4.47)	339 (0.24)
Alcohol use disorder during follow-up, n (%)	631 (2.15)	2,735 (1.93)

BMI, body mass index; MALO, major adverse liver outcome; SDS, standard deviation score.
Continuous variables are presented as median (Q1–Q3), whereas categorical variables are presented as number and proportion (%).
[1]Controls from the general population were matched by sex, birth year, and resident areas.
[2]No liver-related mortality was observed during the follow-up period.

Paediatric obesity was associated with an increased risk of MALOs

During 1,551,994 P–Y of follow-up (median length of follow-up: 8.3 [Q1–Q3 5.5–11.8] years; median age at the end of follow-up: 18.8 [Q1–Q3 15.2–23.0] years), MALOs were observed in 24 individuals in the obesity cohort (IR: 9.0 per 100,000 P–Y) and 53 individuals (IR: 4.1 per 100,000 P–Y) in the general population comparators. The obesity cohort had a higher incidence of MALOs over time (Fig. 1), with an estimated cumulative incidence of 1.14% in the obesity cohort and 0.52% in the general population comparators by age 40 (estimated risk difference: 0.44%, 95% CI 0.32–0.85%). The incidence of liver failure and other cirrhosis-related outcomes, separately, is presented in Fig. S2. Paediatric obesity was associated with an increased risk of MALOs (HR 2.18, 95% CI 1.35–3.53, *p* = 0.001). The risk was similar after adjustment for sex and alcohol use disorder (HR 2.15, 95% CI 1.33–3.48, *p* = 0.002).

Of all individuals developing MALOs in this study (n = 77), the most common diagnosis was ascites (40.3%, n = 31), followed by acute or subacute liver failure (23.4%, n = 18), chronic liver failure (18.2%, n = 14), cirrhosis (10.4%, n = 8), oesophageal or gastric varices (10.4%, n = 8), post-liver transplantation (9.1%, n = 7), and portal hypertension (7.8%, n = 6). No occurrence of hepatocellular carcinoma or liver-related mortality was observed.

Within the obesity cohort, compared to those who did not develop the outcome, the group with MALOs had a higher proportion of class II or III obesity at baseline (66.7% among individuals with MALOs *vs.* 38.9% among those without MALOs) and a lower proportion of obesity remission (8.3% *vs.* 16.6%) (Table S4). Similarly, BMI SDS over time during paediatric years was higher in individuals with MALOs than those without MALOs (in the non-MALO group: average BMI SDS at age 10 years = 2.63 and at age 17 years = 2.64; in MALO group: average BMI SDS at age 10 years = 2.80 and at age 17 years = 3.27) (Fig. S3). Moreover, individuals who had a MASLD diagnosis had a higher incidence of MALOs over time compared to

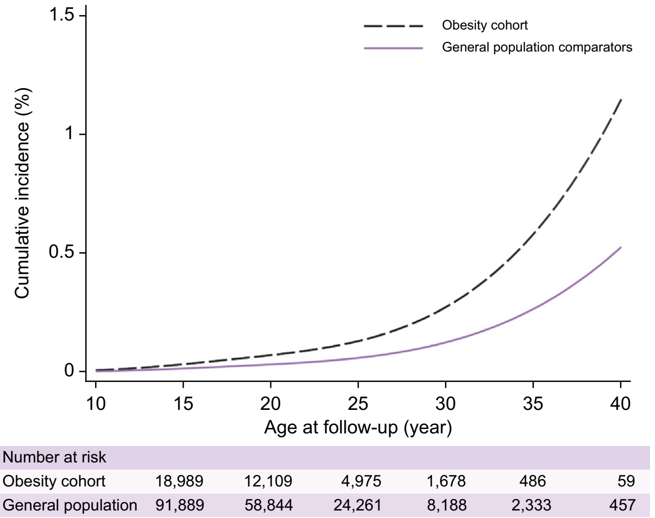

Fig. 1. Cumulative incidence of MALOs between age 10 and 40 years in the obesity cohort and the general population comparators. Cumulative incidence in each group was estimated using a flexible parametric survival model. MALOs, major adverse liver outcomes.

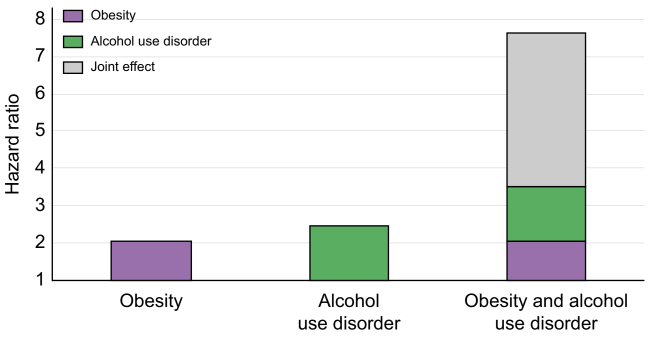

Fig. 2. Joint effect between obesity and alcohol use disorder on the risk of MALOs. Hazard ratios were estimated using Cox regression. The reference group (hazard ratio = 1) was general population comparators without any diagnosis of alcohol use disorders. MALOs, major adverse liver outcomes.

those without a MASLD diagnosis (p <0.001). Likewise, alcohol use disorder in the obesity cohort was also associated with a higher incidence of MALOs (p = 0.019). The incidence of MALOs did not differ by the initial obesity class (p = 0.404). The incidence of MALOs by MASLD, alcohol use disorder, and initial obesity class is shown in Fig. S4.

The joint effect of paediatric obesity and alcohol use disorders during follow-up

Fig. 2 shows HRs of developing MALOs, with the general population without a diagnosis of alcohol use disorder as the reference. The highest HR was observed in the group with obesity and alcohol use disorder (HR 7.64, 95% CI 2.73–21.47, p <0.001). Measures of additive interaction showed a relative excess risk due to interaction of 4.11 (95% CI -3.79 to 12.01, p = 0.31), attributable proportion of 0.54 (95% CI -0.008 to 1.09, p = 0.053), and synergy index of 2.64 (95% CI 0.60–11.59, p = 0.20).

The effect of paediatric obesity on MALOs was not mediated by type 2 diabetes

In the mediation analysis, a direct effect of paediatric obesity on MALOs was observed (HR 2.16, 95% CI 1.09–3.24, p = 0.034), whereas no significant mediating effect of type 2 diabetes was found (indirect effect; HR 1.07, 95% CI -1.15 to 1.30, p = 0.528). The proportion of the association mediated by type 2 diabetes was 6.3% (95% CI -13.8% to 26.3%, p = 0.539).

Sensitivity analysis

The first sensitivity analysis, excluding ascites without any diagnosis of chronic liver disease from the MALO outcome, showed a persistent association between paediatric obesity and MALOs (HR 2.65, 95% CI 1.47–4.79, p = 0.001). In the second sensitivity analysis, adding metabolic bariatric surgery as one of the competing risks, the association between paediatric obesity and MALOs also remained (HR 2.20, 95% CI 1.34–3.59, p = 0.002).

Discussion

This nationwide cohort study demonstrates an association between paediatric obesity and increased risk for MALOs during adolescence and young adulthood. The association was not mediated by type 2 diabetes. Furthermore, a synergistic effect between childhood obesity and alcohol use disorder on MALOs was observed during follow-up.

In line with findings in adult studies,[3,15] we showed that obesity in children and adolescents was associated with double the relative risk for MALOs. While the previous studies[3,15] showed that adult obesity was associated with increased risk in middle and late adulthood, the current study suggests that excess adiposity starting in childhood may contribute to the progression of MALOs and earlier onset of MALO occurrence. However, the double relative risk found in this study should be interpreted carefully given that the absolute risk difference was relatively small.

Despite the relatively low cumulative incidence of MALOs in the obesity cohort during paediatric years, a substantial increasing trend of MALO incidence after age 20 years was observed in the current study. A longer follow-up time would raise the cumulative incidence, given the long-term nature of liver disease progression.[16] Most cases of MALOs during adulthood today occur between age 50 and 80 years.[17] Although less frequent in adolescence and young adulthood, MALOs (for instance, cirrhosis) are generally irreversible, often lead to premature mortality or liver transplantation,[18] and are associated with a high healthcare burden.[19] Moreover, if the prevalence of childhood obesity continues to rise, it is likely that the total number of patients with MALOs will increase and affect healthcare decision-making. Targeted interventions are warranted in patients at a high risk of MALOs. Further studies are crucial to identify subgroups in the paediatric obesity population at the greatest risk of developing MALOs.

The reasons for the positive association between paediatric obesity and the risk of MALOs remain uncertain. A possible explanation is that paediatric obesity is strongly associated with MASLD, which can progress to cirrhosis.[20] Nevertheless, among patients with paediatric obesity who developed MALOs in the present study, about 15% of them had chronic liver diseases other than MASLD, including chronic viral hepatitis, autoimmune hepatitis, primary biliary cholangitis, and alcohol-related liver disease. Hence, it is also possible that low-grade inflammation induced by paediatric obesity[21] may contribute to the progression of chronic liver disease, regardless of the cause. In addition, insulin resistance, linked with paediatric obesity, may promote inflammatory pathways in the liver and lead to hepatic inflammation and apoptosis.[22] Proinflammatory cytokine levels may be elevated further by the crosstalk between the liver and other organs in an environment of excess adiposity.[22] Another explanation is that adult obesity might be a mediator of the association given that paediatric obesity is likely to persist to adulthood.[23] In addition, the current study also showed that alcohol use disorder during follow-up is more common in the obesity cohort than the general population comparators. Although adjustment for alcohol use disorder was performed in analysing the association between paediatric obesity and MALOs, the actual proportion of individuals with excessive alcohol consumption was unknown and could potentially contribute to the association.

The current study suggests a potential synergistic effect of childhood obesity and alcohol use disorders during follow-up on the increased risk of MALOs. Likewise, a Swedish register-based study in adults also found that co-occurrence of MASLD and alcohol use disorder is associated with a 5-fold greater risk of MALOs than MASLD only.[24] In the present study, about 25% of the individuals with alcohol use disorders had already had the diagnosis before 18 years of age. While the traditional approach usually assumes a single cause of liver disease (*e.g.* non-alcohol- *vs.* alcohol-related liver disease), it is recommended to consider mixed causes, especially between metabolic dysfunction and alcohol-related liver disease, even in the paediatric population. The recent international consensus statement defined a new category named MetALD[25] to describe an overlap condition between MASLD and alcohol-related liver disease. The possibility of such an overlapping condition is also emphasized by paediatric hepatology societies.[26] Differentiating solely MASLD from MASLD combined with alcohol use disorder may benefit clinical practice because the latter seems to be associated with a higher risk of developing MALOs. Thus, it is important for paediatricians to assess

alcohol use disorder, especially in caring for adolescents with obesity.

While adult studies have observed type 2 diabetes to be a major contributor, rather than solely obesity, to liver disease progression,[27,28] such a finding was not seen in the current study. The mediation analysis found that the excess risk of MALOs attributable to paediatric obesity was greater than that of type 2 diabetes, indicating that paediatric obesity plays a more important role in the risk of MALOs during adolescence and young adulthood than type 2 diabetes. In addition, we did not observe a significant mediating effect of type 2 diabetes in the association between paediatric obesity and MALOs. A possible explanation is that paediatric obesity generally precedes type 2 diabetes, leading to a longer duration of obesity exposure in the liver. Further, a prolonged duration of type 2 diabetes may also be necessary to affect MALO development.

To our knowledge, this is the first nationwide study with a long follow-up attempting to investigate the effect of childhood obesity on the development of MALOs. The registers used in this study cover the whole nation. MALOs typically require specialised care, and all diagnoses in inpatient and specialised outpatient care in Sweden are recorded in the patient register. Thus, the internal validity of the study is strengthened. However, this study has some limitations. Firstly, data on the underlying cause of MALOs are lacking. Secondly, weight status in the general population comparators and weight trajectories of the obesity cohort after paediatric years were unknown. However, most children in BORIS still had obesity at their last childhood obesity treatment.[29] Future studies incorporating adult weight trajectories are warranted to unravel the mechanism linking paediatric obesity to MALOs. Thirdly, the characteristics of MALOs in this study may not be typical as MALOs usually occur in older age groups. Fourthly, the result of joint effect and mediation analyses might be susceptible to chance findings given the very limited number of individuals with the outcome. Furthermore, the precision and statistical power of the mediation analysis and stratified analyses might be limited due to the small sample size of individuals experiencing the outcome. In addition, the external validity of the findings may be limited to populations with similar demography as Sweden. Different genetic liability, patterns of alcohol consumption and sugar intake between countries may affect the generalisability of the findings. For example, Asian countries, characterised generally by lower alcohol consumption but higher sugar-sweetened beverage intake compared to Western nations, illustrate how dietary factors can affect the risk of MALO.[30,31] Lastly, some cases of liver disease with unspecific symptoms and alcohol use disorder might be underreported in the patient register.

Childhood obesity is associated with an increased risk of MALOs during adolescence and young adulthood. However, relatively few individuals with childhood obesity are affected by MALOs before 40 years of age.

Affiliations

[1]*Department of Clinical Science, Intervention and Technology, Karolinska Institutet, Stockholm, Sweden;* [2]*Department of Medical Epidemiology and Biostatistics, Karolinska Institutet, Stockholm, Sweden*

Abbreviations

BMI, body mass index; BORIS, the Swedish Childhood Obesity Treatment Register; HCC, hepatocellular carcinoma; HR, hazard ratio; ICD, the International Classification of Diseases; IR, incidence rate; MALOs, major adverse liver outcomes; MASLD, metabolic dysfunction associated steatotic liver disease; P–Y, person-years; SDS, standard deviation score.

Financial support

This study was supported by the Freemason Foundation for Children's Welfare inStockholm, the foundation of Sällskapet Barnavård, the HRH Crown Princess Lovisa Society for Child Care, Anna-Lisa & Arne Gustafsson's foundation, The Center for Innovative Medicine (CIMED). The funding sources had no involvement in study design, data analysis, data interpretation, manuscript writing, or the decision to submit the article.

Conflict of interest

PD: Honoraria for lectures: Nestlé; Leadership or fiduciary role on scientific/medical committee: Member of the steering committee for the Swedish Childhood Obesity Treatment Register, Chairman of a working group developing the Swedish national guidelines for paediatric obesity treatment, secretary of the Swedish Childhood Obesity Association. CM: Consulting fees: Novo Nordisk, Rhythm, Oriflame Wellness, DeFaire Medical, Evira AB; Honoraria for lectures: Novo Nordisk, Nestlé, Oriflame Wellness, Astra Zeneca; Payment for expert testimony: Novo Nordic Foundation, Rhythm; Leadership or fiduciary role on scientific/medical committee: board member of ESPE Obesity working group, board member of the Swedish Pediatric Obesity Society, Register holder for the Swedish Childhood Obesity Treatment Register.

EH: Commissioned research for Novo Nordisk (2023), but not for the present study; Honoraria for lectures: Novo Nordisk and Nestlé; Leadership or fiduciary role on scientific/medical committee: Member of the steering committee for the Swedish Childhood Obesity Treatment Register. TC: Member of the working group within Swedish Society of Paediatric Gastroenterology, Hepatology, and Nutrition (SPGHAN) developing the Swedish national guidelines of paediatric MASLD.

RRP had no conflict of interest to disclose.

Please refer to the accompanying ICMJE disclosure forms for further details.

Authors' contributions

Study conception and design: RRP, EH, and CM. Data curation: EH. Data analysis: RRP. Original draft preparation: RRP. TC, PDL, CM, EH, and RRP contributed to the interpretation of the results, provided critical feedback, and approved the final version of the manuscript.

Data availability statement

Patient-level data cannot be shared publicly because of third-party data. Given that an ethical approval is obtained, any individual may apply for data from Statistics Sweden via information@scb.se, the Swedish National Board of Health and Welfare via registerservice@socialstyrelsen.se, and the Swedish Childhood Obesity Treatment Register via http://www.e-boris.se/in-english/.

Acknowledgements

The authors would like to thank all the paediatric obesity treatment units for their contributions to the BORIS register.

Supplementary data

Supplementary data to this article can be found online at https://doi.org/10.1016/j.jhepr.2025.101425.

References

[1] Li J, Ha A, Rui F, et al. Meta-analysis: global prevalence, trend and forecasting of non-alcoholic fatty liver disease in children and adolescents, 2000–2021. Aliment Pharmacol Ther 2022;56:396–406.

[2] Panganiban J, Kehar M, Ibrahim SH, et al. Metabolic dysfunction-associated steatotic liver disease (MASLD) in children with obesity: an obesity medicine

association (OMA) and expert joint perspective 2025. Obes Pillars 2025:100164.
[3] Hagström H, Tynelius P, Rasmussen F. High BMI in late adolescence predicts future severe liver disease and hepatocellular carcinoma: a national, population-based cohort study in 1.2 million men. Gut 2018;67:1536–1542.
[4] Berzigotti A, Garcia-Tsao G, Bosch J, et al. Obesity is an independent risk factor for clinical decompensation in patients with cirrhosis. Hepatology 2011;54:555–561.
[5] Cole TJ, Lobstein T. Extended international (IOTF) body mass index cut-offs for thinness, overweight and obesity. Pediatr Obes 2012;7:284–294.
[6] Bervoets L, Massa G. Defining morbid obesity in children based on BMI 40 at age 18 using the extended international (IOTF) cut-offs. Pediatr Obes 2014;9:e94–e98.
[7] Ioannou GN, Weiss NS, Kowdley KV, et al. Is obesity a risk factor for cirrhosis-related death or hospitalization? A population-based cohort study. Gastroenterology 2003;125:1053–1059.
[8] Putri RR, Casswall T, Danielsson P, et al. Steatotic liver disease in pediatric obesity and increased risk for youth-onset type 2 diabetes. Diabetes Care 2024;47(12):2196-2204.
[9] Wang G, Huang Y, Yang H, et al. Impacts of bariatric surgery on adverse liver outcomes: a systematic review and meta-analysis. Surg Obes Relat Dis 2023;19:717–726.
[10] Hagman E, Danielsson P, Lindberg L, et al. Paediatric obesity treatment during 14 years in Sweden: lessons from the Swedish childhood obesity treatment register—BORIS. Pediatr Obes 2020;15:1–9.
[11] Ludvigsson JF, Andersson E, Ekbom A, et al. External review and validation of the Swedish national inpatient register. BMC Public Health 2011;11:450.
[12] Bengtsson B, Askling J, Ludvigsson JF, et al. Validity of administrative codes associated with cirrhosis in Sweden. Scand J Gastroenterol 2020;55:1205–1210.
[13] Andersson T, Alfredsson L, Källberg H, et al. Calculating measures of biological interaction. Eur J Epidemiol 2005;20:575–579.
[14] VanderWeele TJ. Mediation analysis: a practitioner's guide. Annu Rev Public Health 2016;37:17–32.
[15] De Vincentis A, Tavaglione F, Spagnuolo R, et al. Metabolic and genetic determinants for progression to severe liver disease in subjects with obesity from the UK Biobank. Int J Obes 2022;46:486–493.
[16] Tajiri K, Shimizu Y. Liver physiology and liver diseases in the elderly. World J Gastroenterol 2013;19:8459–8467.
[17] Vaz J, Eriksson B, Strömberg U, et al. Incidence, aetiology and related comorbidities of cirrhosis: a Swedish population-based cohort study. BMC Gastroenterol 2020;20:84.
[18] Bajaj JS, Choudhury AK, Xie Q, et al. Global disparities in mortality and liver transplantation in hospitalised patients with cirrhosis: a prospective cohort study for the CLEARED Consortium. Lancet Gastroenterol Hepatol 2023;8:611–622.
[19] Lau NSM, Henderson P. Outcomes of paediatric patients with chronic liver disease in early adulthood: a heterogeneous, but representative, regional cohort study. J Paediatr Child Health 2022;58:1771–1777.
[20] Perumpail BJ, Manikat R, Wijarnpreecha K, et al. The prevalence and predictors of metabolic dysfunction-associated steatotic liver disease and fibrosis/cirrhosis among adolescents/young adults. J Pediatr Gastroenterol Nutr 2024:1–9.
[21] Marcus C, Danielsson P, Hagman E. Pediatric obesity—long-term consequences and effect of weight loss. J Intern Med 2022;292:870–891.
[22] Gehrke N, Schattenberg JM. Metabolic inflammation—a role for hepatic inflammatory pathways as drivers of comorbidities in nonalcoholic fatty liver disease? Gastroenterology 2020;158:1929–1947.e6.
[23] Ward ZJ, Long MW, Resch SC, et al. Simulation of growth trajectories of childhood obesity into adulthood. New Engl J Med 2017;377:2145–2153.
[24] Nasr P, Wester A, Ekstedt M, et al. Misclassified alcohol-related liver disease is common in presumed metabolic dysfunction-associated steatotic liver disease and highly increases risk for future cirrhosis. Clin Gastroenterol Hepatol 2024;22:1048–1057.e2.
[25] Rinella ME, Lazarus JV, Ratziu V, et al. A multisociety Delphi consensus statement on new fatty liver disease nomenclature. J Hepatol 2023;79:1542–1556.
[26] Baumann U, Koot BGP, Fitzpatrick E, et al. Paediatric steatotic liver disease has unique characteristics: a multisociety statement endorsing the new nomenclature. J Pediatr Gastroenterol Nutr 2024;78:1190–1196.
[27] Mittal N, Siddiqi H, Madamba E, et al. A prospective study on the prevalence of at-risk MASH in patients with type 2 diabetes mellitus in the United States. Aliment Pharmacol Ther 2024;59:1571–1578.
[28] Jarvis H, Craig D, Barker R, et al. Metabolic risk factors and incident advanced liver disease in non-alcoholic fatty liver disease (NAFLD): a systematic review and meta-analysis of population-based observational studies. Plos Med 2020;17:e1003100.
[29] The Swedish Childhood Obesity Treatment Register. BORIS annual report. 2022.
[30] Huang DQ, Terrault NA, Tacke F, et al. Global epidemiology of cirrhosis — aetiology, trends and predictions. Nat Rev Gastroenterol Hepatol 2023;20:388–398.
[31] Miller V, Webb P, Cudhea F, et al. Global dietary quality in 185 countries from 1990 to 2018 show wide differences by nation, age, education, and urbanicity. Nat Food 2022;3:694–702.

Keywords: obesity; paediatric obesity; liver failure; cirrhosis; alcohol use disorder; type 2 diabetes.
Received 16 December 2024; received in revised form 31 March 2025; accepted 7 April 2025; Available online 11 April 2025

Journal of Hepatology, Volume *7*

Supplemental information

The association between childhood obesity and major adverse liver outcomes in adolescence and young adulthood

Resthie R. Putri, Thomas Casswall, Pernilla Danielsson, Claude Marcus, and Emilia Hagman

The association between childhood obesity and major adverse liver outcomes in adolescence and young adulthood

Resthie R Putri, Thomas Casswall, Pernilla Danielsson, Claude Marcus, Emilia Hagman

Table of contents

Table S1……………………………………………………………………………..2

Table S2……………………………………………………………………………..3

Table S3……………………………………………………………………………..4

Table S4……………………………………………………………………………..5

Fig. S1………………………………………………………………………………6

Fig. S2………………………………………………………………………………8

Fig. S3………………………………………………………………………………9

Fig. S4……………………………………………………………………………...10

Table S1. ICD-10 codes for the outcome of major adverse liver outcomes (MALO) and the exclusion criteria of genetic syndromes

ICD-10 code	Diagnosis
MALO	
K74.6	Cirrhosis
K76.6	Portal hypertension
I86.4	Gastric varices
I85.0, I85.9, I98.2, I98.3	Oesophageal varices
R18	Ascites
K72.0, K72.1, K72.9	Liver failure
C22.0	Hepatocellular carcinoma
Z94.4	Liver transplantation
Genetic syndromes	
Q44.7	Alagille syndrome
Q90	Down syndrome
Q99.2	Fragile X syndrome
Q98	Klinefelter syndrome
Q87.8B	Laurence-Moon-Biedl syndrome
Q87.1E	Noonan syndrome
Q87.1F	Prader-Willi syndrome
Q87.1G	Silver-Russel syndrome
Q96	Turner syndrome

Table S2. ICD-10 codes for alcohol use disorder

ICD-10 code	Diagnosis
E24.4	Alcohol-induced pseudo-Cushing's syndrome
F10	Mental and behavioural disorders due to use of alcohol
G62.1	Alcoholic polyneuropathy
I42.6	Alcoholic cardiomyopathy
K29.2	Alcoholic gastritis
G31.2	Degeneration of nervous system due to alcohol
G72.1	Alcoholic myopathy
K70	Alcoholic liver disease
K85.2	Alcohol-induced acute pancreatitis
K86.0	Alcohol-induced chronic pancreatitis
T51	Toxic effect of alcohol

Table S3. Codes for diagnosis of type 2 diabetes, antidiabetic medications, and volume-restricting bariatric surgery

Data sources	Codes
Type 2 diabetes	
The Swedish National Patient Register (based on ICD-10 codes)	E11. Type 2 diabetes<br>O24.1. Pre-existing type 2 diabetes mellitus, in pregnancy, childbirth and the puerperium
The Prescribed Drug Register (based on Anatomical Therapeutic Chemical codes)	A10BA biguanides<br>A10BB sulfonylureas<br>A10BC sulfonamides<br>A10BD antidiabetic combinations<br>A10BF alpha glucosidase inhibitors<br>A10BG thiazolidinediones<br>A10BH DPP4 inhibitors<br>A10BJ GLP-1 analogues<br>A10BK SGLT2 inhibitors<br>Other antidiabetic A10BX<br>Insulin A10A
Metabolic bariatric surgery	
National Patient Register	Procedure code for volume-restricting bariatric surgery (JDF) in combination with ICD-10 code for obesity (E66).

Table S4. Characteristics of patients in the paediatric obesity cohort based on the event outcome occurrence of MALO

	No MALO (N = 28 287)	MALO (N = 24)
Age at treatment initiation, median (Q1, Q3)	10.5 (8.1, 13.2)	13.0 (9.2, 16.6)
Overweight or class I obesity, n (%)	17 273 (61.1)	8 (33.3)
Class II or III obesity, n (%)	11 014 (38.9)	16 (66.7)
Change in BMI SDS[1], median (Q1, Q3)	-0.06 (-0.31, 0)	-0.04 (-0.27, 0)
Stay in the same obesity class at the last visit of obesity treatment, n (%)	11 747 (68.4)	13 (68.4)
Obesity remission at the last visit of obesity treatment, n (%)	4692 (16.6)	2 (8.3)
Obesity treatment duration, median (Q1, Q3)	17.3 (3.3 – 41.0)	23.6 (8.4 – 54.9)
Any recorded ALT >35 U/L during obesity treatment[2], n (%)	4632 (25.2)	7 (36.9)
Any recorded fasting glucose ≥6.1 mmol/L during obesity treatment[3], n (%)	979 (5.5)	2 (10.5)

Abbreviations: ALT, alanine aminotransferases; BMI SDS, body mass index standard deviation score, Q1, quartile 1; Q3, quartile 3

[1]BMI SDS reduction was calculated among individuals who had more than 1 visit in obesity treatment as BMI SDS at the last visit – BMI SDS at the first visit.

[2]Among those who had at least one ALT data in the paediatric obesity register (n = 19 in the MALO group and n = 18395 in the non-MALO group)

[3]Among those who had at least one fasting glucose data in the paediatric obesity register (n = 19 in the MALO group and n = 17926 in the non-MALO group)

Fig. S1. Algorithm to ascertain type 2 diabetes

The algorithm is divided into three groups:

- Algorithm A: Individuals who had diagnosis of type 2 diabetes in specialized care (i.e., recorded in the Patient Register) but did not receive prescribed antidiabetic medications.
- Algorithm B: Individuals who received prescribed antidiabetic medications but did not have recorded diagnosis of type 2 diabetes in specialized care.
- Algorithm C: Individuals who both received antidiabetic medications and had recorded diagnosis of type 2 diabetes in specialized care.

Algorithm A: Diagnosis of type 2 diabetes in specialized care = yes & any antidiabetic medications = no

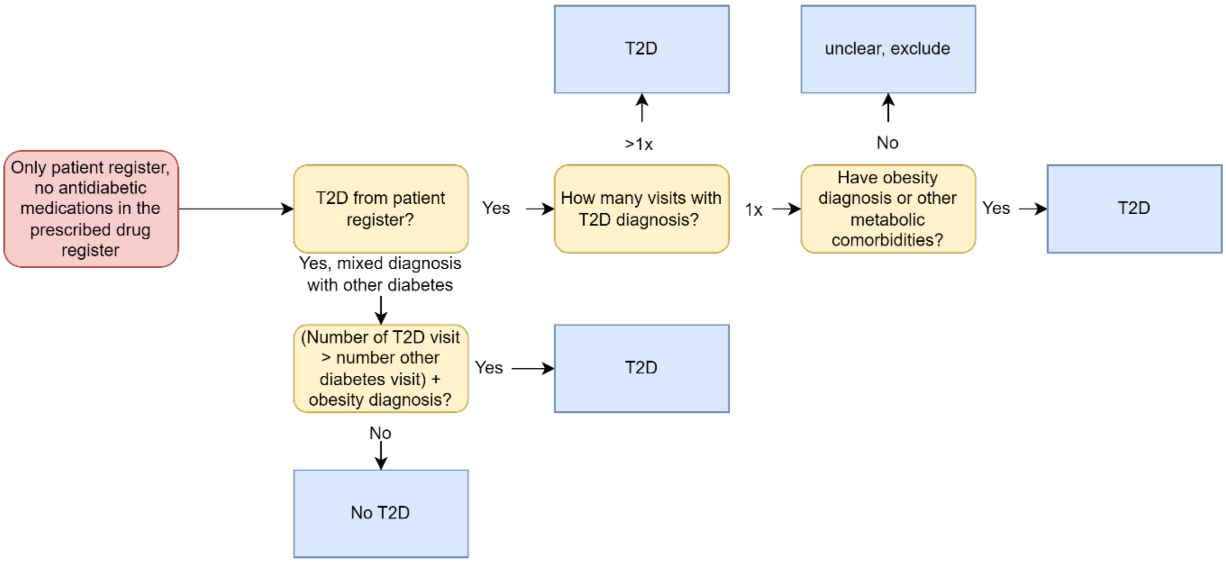

Algorithm B: Diagnosis of type 2 diabetes in specialized care = no & any antidiabetic medications = yes

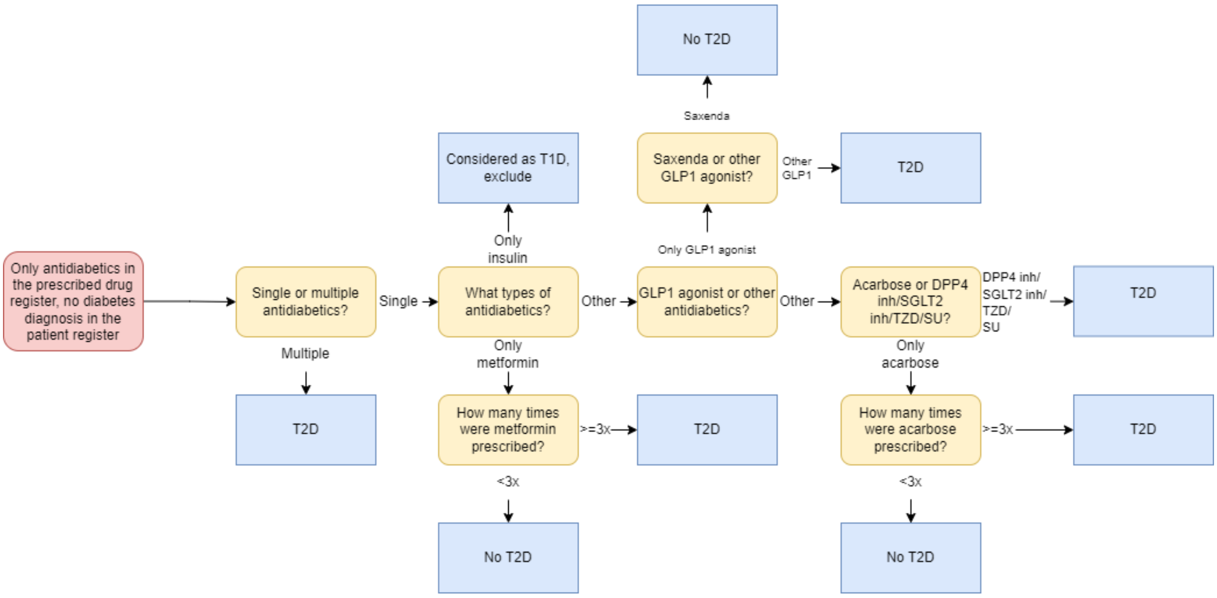

Algorithm C: Diagnosis of type 2 diabetes in specialized care = yes & any antidiabetic medications = yes

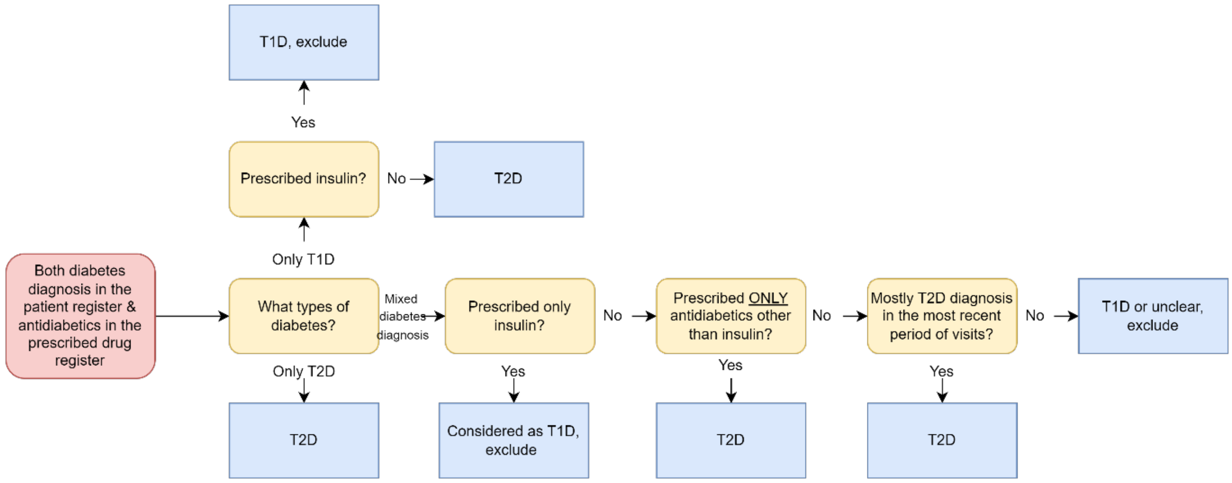

Fig. S2. Higher cumulative incidence of MALO (cirrhosis and liver failure, separately) in the paediatric obesity cohort and general population comparators

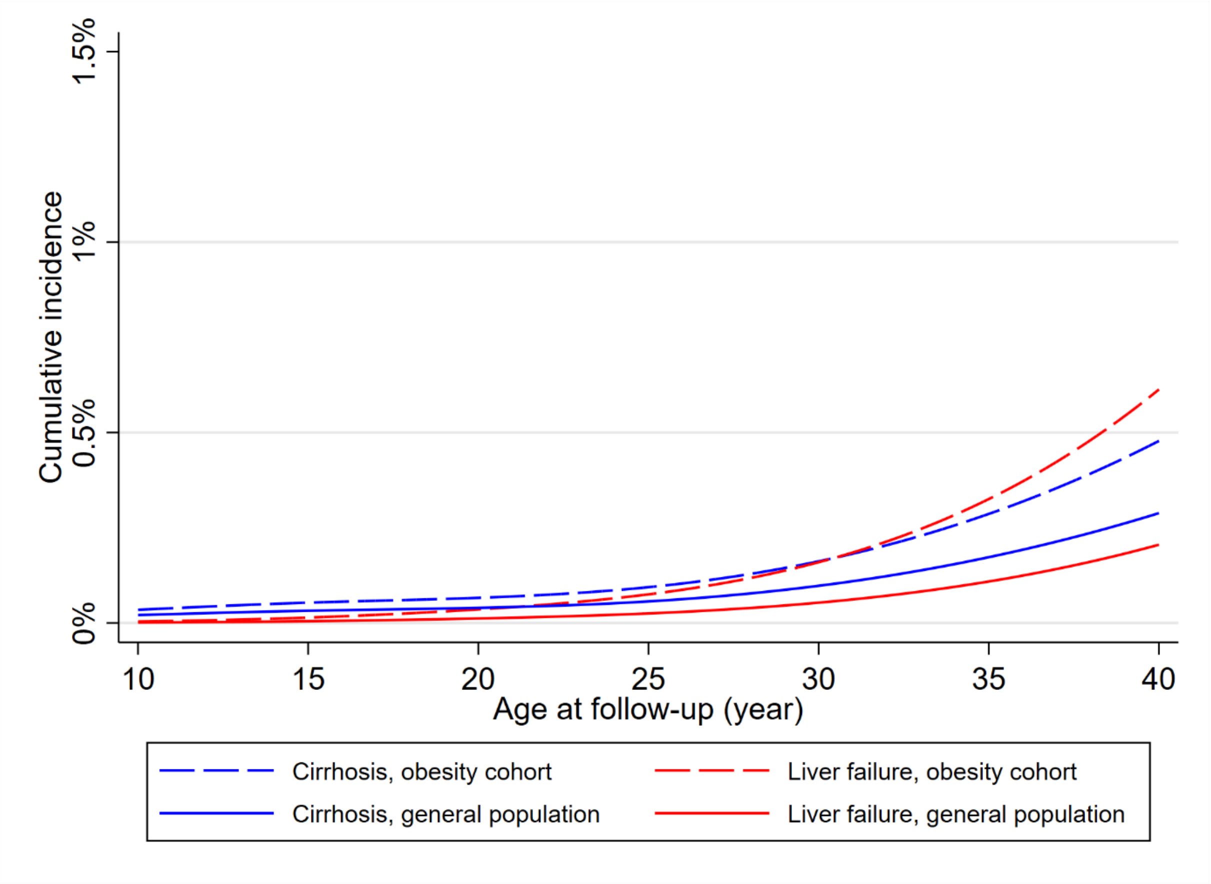

Estimated cumulative incidence of cirrhosis and liver failure was calculated based on flexible parametric models with competing risks with three degrees of freedom. Cirrhosis was defined based on all MALO related to decompensated cirrhosis (i.e., cirrhosis, oesophageal varices, gastric varices, portal hypertension, ascites). Liver failure was defined based on diagnosis of acute, sub-acute, or chronic liver failure.

Fig. S3. Longitudinal change of BMI SDS in the paediatric obesity cohort

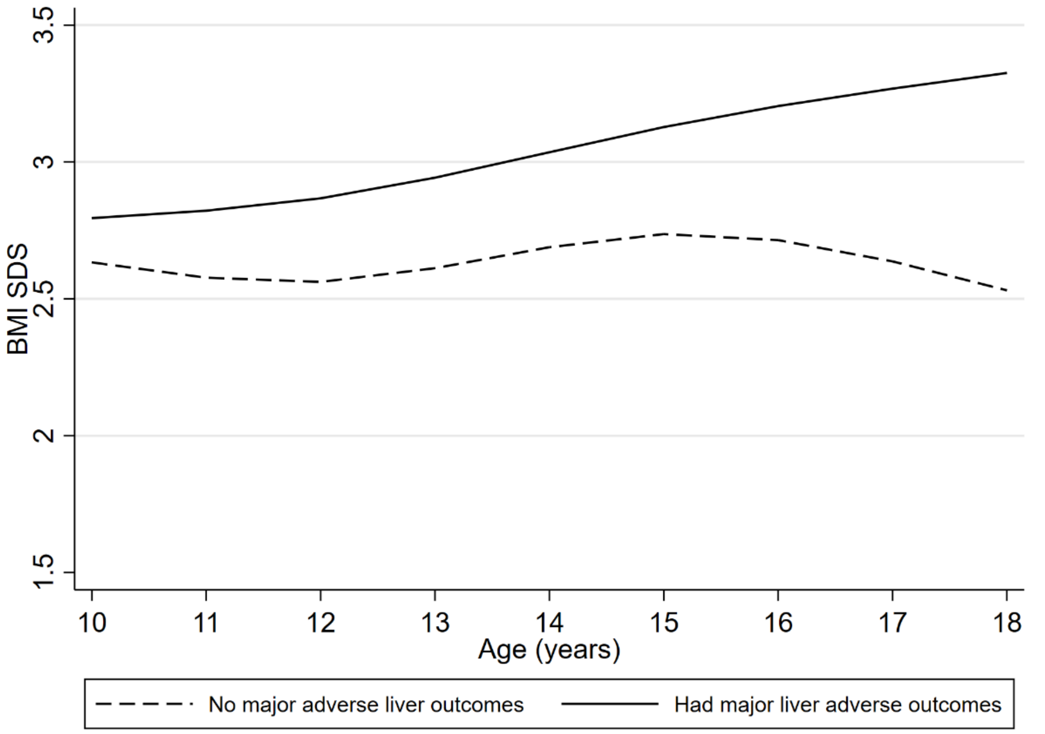

Y-axis represents estimated average BMI SDS. X-axis represents age (years). The longitudinal change of average BMI SDS over time was estimated using linear mixed model.

Fig. S4. Incidence of MALO within the obesity cohort, divided by diagnosis of MASLD, alcohol use disorder, and initial obesity class

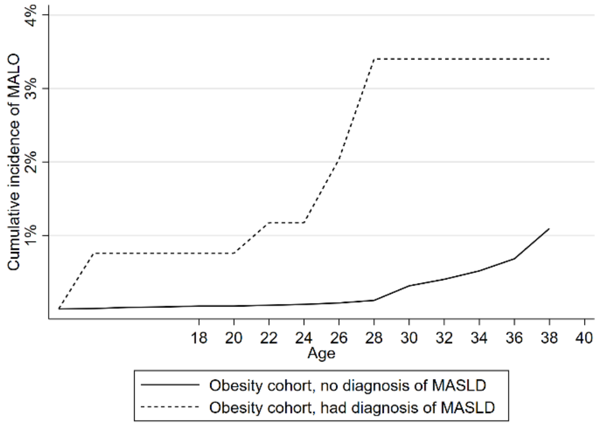

Figure S8 a) Incidence of MALO in the obesity cohort divided by diagnosis of MASLD, based on Kaplan Meier estimates (P log-rank <0.001).

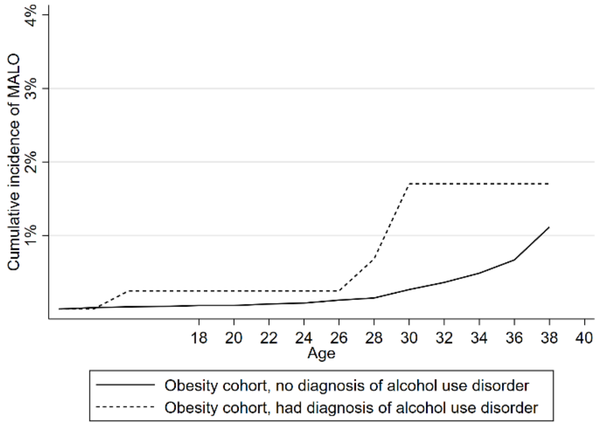

Figure S8 b) Incidence of MALO in the obesity cohort divided by diagnosis of alcohol use disorder, based on Kaplan Meier estimates (P log-rank = 0.019).

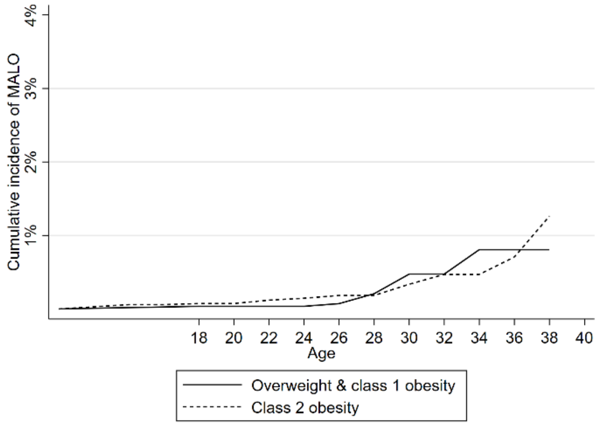

Figure S8 c) Incidence of MALO in the obesity cohort divided by initial degree of obesity, based on Kaplan Meier estimates (P log-rank = 0.404).
